# 1,2,3,4-Tetra­hydro-1,4-methano­naphthalene-2,3-diol

**DOI:** 10.1107/S1600536808041068

**Published:** 2008-12-10

**Authors:** Jian Xu, Hao Xu, Ji-cai Quan, Fei Sha, Cheng Yao

**Affiliations:** a College of Science, Nanjing University of Technology, Xinmofan Road No. 5, Nanjing 210009, People’s Republic of China

## Abstract

The title compound, C_11_H_12_O_2_, is an inter­mediate in the synthesis of Varenicline, a nicotinic receptor partial agonist used to treat smoking addiction. In the crystal structure, there is an intra­molecular O—H⋯O hydrogen bond that generates an *S*(5) ring motif. Inter­molecular O—H⋯O hydrogen bonds form centrosymmetric dimers and also link these dimers into chains along the *a* axis.

## Related literature

For background to the use of Varenicline to treat smoking addiction, see: Vetelino, (2004 ▶); Coe (2005 ▶). For details of graph-set analysis of hydrogen-bonding patterns, see: Bernstein *et al.* (1995 ▶).
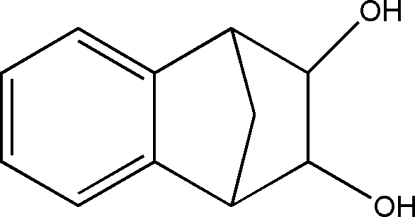


## Experimental

### 

#### Crystal data

C_11_H_12_O_2_
*M* *_r_* = 176.21
Orthorhombic, 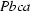

*a* = 10.240 (2) Å
*b* = 6.2370 (12) Å
*c* = 27.503 (6) Å
*V* = 1756.5 (6) Å^3^
*Z* = 8
Mo *K*α radiation
μ = 0.09 mm^−1^
*T* = 293 (2) K
0.30 × 0.20 × 0.10 mm

#### Data collection

Enraf–Nonius CAD-4 diffractometer
Absorption correction: ψ scan (North *et al.*, 1968 ▶) *T*_min_ = 0.973, *T*_max_ = 0.991
1581 measured reflections
1581 independent reflections
1045 reflections with *I* > 2σ(*I*)
3 standard reflections every 200 reflections intensity decay: none

#### Refinement

*R*[*F* ^2^ > 2σ(*F* ^2^)] = 0.063
*wR*(*F* ^2^) = 0.168
*S* = 1.03
1581 reflections
118 parameters
H-atom parameters constrained
Δρ_max_ = 0.24 e Å^−3^
Δρ_min_ = −0.27 e Å^−3^

### 

Data collection: *CAD-4 Software* (Enraf–Nonius, 1989 ▶); cell refinement: *CAD-4 Software*; data reduction: *XCAD4* (Harms & Wocadlo, 1995 ▶); program(s) used to solve structure: *SHELXS97* (Sheldrick, 2008 ▶); program(s) used to refine structure: *SHELXL97* (Sheldrick, 2008 ▶); molecular graphics: *SHELXTL* (Sheldrick, 2008 ▶); software used to prepare material for publication: *SHELXL97*.

## Supplementary Material

Crystal structure: contains datablocks global, I. DOI: 10.1107/S1600536808041068/sj2556sup1.cif

Structure factors: contains datablocks I. DOI: 10.1107/S1600536808041068/sj2556Isup2.hkl

Additional supplementary materials:  crystallographic information; 3D view; checkCIF report

## Figures and Tables

**Table 1:** Hydrogen-bond geometry (Å, °)

*D*—H⋯*A*	*D*—H	H⋯*A*	*D*⋯*A*	*D*—H⋯*A*
O1—H1*A*⋯O2	0.85	2.16	2.578 (3)	110
O1—H1*A*⋯O2^i^	0.85	2.34	2.818 (3)	116
O2—H2*A*⋯O1^ii^	0.82	1.90	2.714 (3)	176

Symmetry codes: (i) 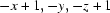; (ii) 
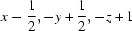
.

**Fig. 1.**
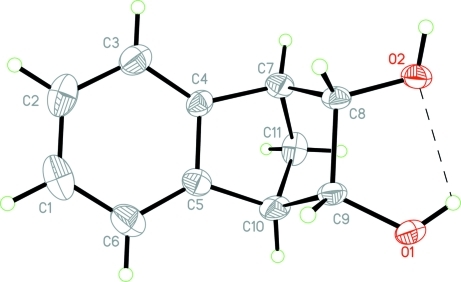
The molecular structure of (I), showing the atom-numbering scheme and displacement ellipsoids at the 30% probability level.

**Fig. 2.**
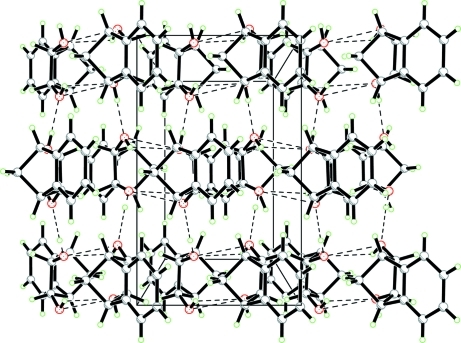
Crystal packing of (I) viewed down the c axis with hydrogen bonds drawn as dashed lines.

## supplementary crystallographic information

### Comment

The title compound, I, is an important intermediate in the synthesis of Varenicline, a nicotinic receptor partial agonist used to treat smoking addiction (Vetelino, 2004). Varenicline came onto the market in 2006 and displays high affinity for neuronal nicotinic acetylcholine receptors (nAChRs), which mediate the dependence-producing effects of nicotine (Coe, 2005).

We report here the crystal structure of the title compound, (I), Fig. 1. The saturated six-membered C4,C5,C7···C10 ring of the anthracene group carries hydroxy substituents on C8 and C9 and is bridged by the C11 methylene group. In the crystal structure an intramolecular O1—H1A···O2 hydrogen bond generates an S5 ring motif (Bernstein *et al.*, 1995). Intermolecular O1—H1A···O2 hydrogen bonds form centrosymmetric dimers and link these dimers into chains along the *a* axis, Table 2, Figure 2.

### Experimental

1,4-Dihydro-1,4-methanonaphthalene (79.5 g, 560 mmol) in acetone (800 ml) and H_2_O (100 ml) was stirred with *N*-methyl morpholine N-oxide (67.5 g, 576 mmol). OsO4 (15 ml of a 15 mol% t-BuOH solution, 1.48 mmol,0.26mol%) was added and the mixture was stirred vigorously. After 60 h, the solution was filtered, and the white solid product rinsed with acetone and air-dried (60.9 g). The mother liquor was partially concentrated to an oily solid which was triturated with acetone, filtered and rinsed with acetone to provide additional amounts of the title compound (27.4 g, total 88.3 g, 89%). An X-ray grade crystal of I was grown from acetone (10 ml) at room temperature.

### Refinement

H atoms bound to O were located in a difference Fourier map and fixed in these positions with U_iso_ = 1.5U_eq_ (O). Other H-atoms were positioned geometrically and refined using a riding model with d(C-H) = 0.93Å, U_iso_ = 1.2U_eq_ (C) for aromatic 0.98Å, U_iso_ = 1.2U_eq_ (C) for CH, 0.97Å, U_iso_ = 1.2U_eq_ (C) for CH_2_ groups.

### Crystal data

**Table:**

C_11_H_12_O_2_	*F*(000) = 752
*M**_r_* = 176.21	*D*_x_ = 1.333 Mg m^−^^3^
Orthorhombic, *P**b**c**a*	Mo *K*α radiation, λ = 0.71073 Å
Hall symbol: -P 2ac 2ab	Cell parameters from 25 reflections
*a* = 10.240 (2) Å	θ = 10–13°
*b* = 6.2370 (12) Å	µ = 0.09 mm^−^^1^
*c* = 27.503 (6) Å	*T* = 293 K
*V* = 1756.5 (6) Å^3^	White, colourless
*Z* = 8	0.30 × 0.20 × 0.10 mm

### Data collection

**Table:**

Enraf–Nonius CAD-4 diffractometer	1045 reflections with *I* > 2σ(*I*)
Radiation source: fine-focus sealed tube	*R*_int_ = 0.0000
graphite	θ_max_ = 25.2°, θ_min_ = 1.5°
ω/2θ scans	*h* = 0→12
Absorption correction: ψ scan (North *et al.*, 1968)	*k* = 0→7
*T*_min_ = 0.973, *T*_max_ = 0.991	*l* = 0→32
1581 measured reflections	3 standard reflections every 200 reflections
1581 independent reflections	intensity decay: none

### Refinement

**Table:**

Refinement on *F*^2^	Primary atom site location: structure-invariant direct methods
Least-squares matrix: full	Secondary atom site location: difference Fourier map
*R*[*F*^2^ > 2σ(*F*^2^)] = 0.063	Hydrogen site location: inferred from neighbouring sites
*wR*(*F*^2^) = 0.168	H-atom parameters constrained
*S* = 1.02	*w* = 1/[σ^2^(*F*_o_^2^) + (0.06*P*)^2^ + 3*P*] where *P* = (*F*_o_^2^ + 2*F*_c_^2^)/3
1581 reflections	(Δ/σ)_max_ < 0.001
118 parameters	Δρ_max_ = 0.24 e Å^−^^3^
0 restraints	Δρ_min_ = −0.27 e Å^−^^3^

### Special details

**Table:**

Geometry. All e.s.d.'s (except the e.s.d. in the dihedral angle between two l.s. planes) are estimated using the full covariance matrix. The cell e.s.d.'s are taken into account individually in the estimation of e.s.d.'s in distances, angles and torsion angles; correlations between e.s.d.'s in cell parameters are only used when they are defined by crystal symmetry. An approximate (isotropic) treatment of cell e.s.d.'s is used for estimating e.s.d.'s involving l.s. planes.
Refinement. Refinement of *F*^2^ against ALL reflections. The weighted *R*-factor *wR* and goodness of fit *S* are based on *F*^2^, conventional *R*-factors *R* are based on *F*, with *F* set to zero for negative *F*^2^. The threshold expression of *F*^2^ > σ(*F*^2^) is used only for calculating *R*-factors(gt) *etc*. and is not relevant to the choice of reflections for refinement. *R*-factors based on *F*^2^ are statistically about twice as large as those based on *F*, and *R*- factors based on ALL data will be even larger.

### Fractional atomic coordinates and isotropic or equivalent isotropic displacement parameters (Å^2^)

**Table:**

	*x*	*y*	*z*	*U*_iso_*/*U*_eq_	
O1	0.65327 (18)	0.2295 (4)	0.51706 (7)	0.0289 (5)	
H1A	0.6083	0.1798	0.4936	0.035*	
C1	0.5544 (4)	0.5675 (6)	0.71478 (12)	0.0417 (9)	
H1B	0.5950	0.6468	0.7391	0.050*	
O2	0.40283 (18)	0.1947 (3)	0.51141 (7)	0.0287 (6)	
H2A	0.3270	0.2214	0.5040	0.043*	
C2	0.4181 (4)	0.5663 (6)	0.71136 (12)	0.0392 (9)	
H2B	0.3686	0.6463	0.7332	0.047*	
C3	0.3563 (3)	0.4465 (5)	0.67558 (11)	0.0333 (8)	
H3A	0.2657	0.4456	0.6733	0.040*	
C4	0.4303 (3)	0.3299 (5)	0.64387 (10)	0.0235 (7)	
C5	0.5676 (3)	0.3324 (5)	0.64679 (10)	0.0258 (7)	
C6	0.6296 (3)	0.4508 (6)	0.68204 (11)	0.0359 (8)	
H6A	0.7203	0.4529	0.6840	0.043*	
C7	0.3981 (3)	0.1886 (5)	0.60039 (10)	0.0260 (7)	
H7A	0.3096	0.1286	0.6003	0.031*	
C8	0.4366 (2)	0.3095 (5)	0.55428 (10)	0.0202 (6)	
H8A	0.3991	0.4540	0.5540	0.024*	
C9	0.5897 (2)	0.3180 (5)	0.55809 (10)	0.0214 (7)	
H9A	0.6174	0.4673	0.5621	0.026*	
C10	0.6168 (3)	0.1942 (5)	0.60542 (10)	0.0281 (8)	
H10A	0.7059	0.1395	0.6091	0.034*	
C11	0.5087 (3)	0.0226 (5)	0.60427 (11)	0.0313 (8)	
H11A	0.5142	−0.0706	0.5761	0.038*	
H11B	0.5046	−0.0617	0.6339	0.038*	

### Atomic displacement parameters (Å^2^)

**Table:**

	*U*^11^	*U*^22^	*U*^33^	*U*^12^	*U*^13^	*U*^23^
O1	0.0170 (9)	0.0410 (13)	0.0288 (11)	−0.0049 (10)	0.0050 (9)	−0.0077 (10)
C1	0.062 (2)	0.034 (2)	0.0297 (18)	−0.0094 (19)	−0.0103 (17)	−0.0012 (16)
O2	0.0185 (10)	0.0364 (13)	0.0311 (12)	0.0070 (10)	−0.0050 (9)	−0.0120 (10)
C2	0.056 (2)	0.034 (2)	0.0277 (18)	0.0070 (18)	0.0077 (16)	−0.0010 (15)
C3	0.0305 (17)	0.0378 (19)	0.0315 (17)	0.0057 (16)	0.0044 (14)	0.0014 (15)
C4	0.0233 (15)	0.0265 (17)	0.0206 (15)	0.0022 (13)	0.0038 (12)	0.0031 (13)
C5	0.0254 (15)	0.0266 (17)	0.0255 (16)	0.0016 (13)	−0.0039 (12)	0.0037 (13)
C6	0.0338 (18)	0.041 (2)	0.0324 (18)	−0.0073 (17)	−0.0066 (15)	0.0035 (16)
C7	0.0203 (14)	0.0264 (17)	0.0315 (17)	−0.0076 (13)	0.0012 (12)	−0.0020 (14)
C8	0.0156 (14)	0.0181 (15)	0.0270 (15)	0.0034 (12)	−0.0020 (12)	−0.0022 (13)
C9	0.0146 (13)	0.0233 (15)	0.0263 (15)	−0.0023 (12)	0.0022 (12)	−0.0024 (13)
C10	0.0200 (15)	0.0345 (19)	0.0297 (16)	0.0114 (14)	−0.0018 (12)	0.0050 (15)
C11	0.0453 (19)	0.0222 (16)	0.0265 (16)	0.0034 (15)	0.0021 (14)	0.0030 (14)

### Geometric parameters (Å, °)

**Table:**

O1—C9	1.415 (3)	C5—C10	1.513 (4)
O1—H1A	0.8501	C6—H6A	0.9300
C1—C6	1.390 (5)	C7—C8	1.527 (4)
C1—C2	1.399 (5)	C7—C11	1.538 (4)
C1—H1B	0.9300	C7—H7A	0.9800
O2—C8	1.422 (3)	C8—C9	1.572 (4)
O2—H2A	0.8200	C8—H8A	0.9800
C2—C3	1.388 (5)	C9—C10	1.539 (4)
C2—H2B	0.9300	C9—H9A	0.9800
C3—C4	1.365 (4)	C10—C11	1.540 (4)
C3—H3A	0.9300	C10—H10A	0.9800
C4—C5	1.408 (4)	C11—H11A	0.9700
C4—C7	1.522 (4)	C11—H11B	0.9700
C5—C6	1.374 (4)		
			
C9—O1—H1A	119.8	C11—C7—H7A	115.1
C6—C1—C2	120.4 (3)	O2—C8—C7	112.1 (2)
C6—C1—H1B	119.8	O2—C8—C9	108.3 (2)
C2—C1—H1B	119.8	C7—C8—C9	102.6 (2)
C8—O2—H2A	109.5	O2—C8—H8A	111.1
C3—C2—C1	120.3 (3)	C7—C8—H8A	111.1
C3—C2—H2B	119.8	C9—C8—H8A	111.1
C1—C2—H2B	119.8	O1—C9—C10	113.4 (2)
C4—C3—C2	119.1 (3)	O1—C9—C8	113.1 (2)
C4—C3—H3A	120.4	C10—C9—C8	102.6 (2)
C2—C3—H3A	120.4	O1—C9—H9A	109.2
C3—C4—C5	120.8 (3)	C10—C9—H9A	109.2
C3—C4—C7	133.6 (3)	C8—C9—H9A	109.2
C5—C4—C7	105.5 (2)	C5—C10—C9	106.9 (2)
C6—C5—C4	120.5 (3)	C5—C10—C11	99.9 (2)
C6—C5—C10	133.1 (3)	C9—C10—C11	101.7 (2)
C4—C5—C10	106.4 (3)	C5—C10—H10A	115.5
C5—C6—C1	118.9 (3)	C9—C10—H10A	115.5
C5—C6—H6A	120.6	C11—C10—H10A	115.5
C1—C6—H6A	120.6	C7—C11—C10	93.6 (2)
C4—C7—C8	108.1 (2)	C7—C11—H11A	113.0
C4—C7—C11	100.1 (2)	C10—C11—H11A	113.0
C8—C7—C11	101.5 (2)	C7—C11—H11B	113.0
C4—C7—H7A	115.1	C10—C11—H11B	113.0
C8—C7—H7A	115.1	H11A—C11—H11B	110.4
			
C6—C1—C2—C3	0.8 (5)	C11—C7—C8—C9	−37.4 (3)
C1—C2—C3—C4	0.0 (5)	O2—C8—C9—O1	5.3 (3)
C2—C3—C4—C5	−0.7 (5)	C7—C8—C9—O1	124.1 (2)
C2—C3—C4—C7	−178.0 (3)	O2—C8—C9—C10	−117.2 (2)
C3—C4—C5—C6	0.7 (5)	C7—C8—C9—C10	1.5 (3)
C7—C4—C5—C6	178.6 (3)	C6—C5—C10—C9	−107.6 (4)
C3—C4—C5—C10	−177.9 (3)	C4—C5—C10—C9	70.6 (3)
C7—C4—C5—C10	0.1 (3)	C6—C5—C10—C11	146.8 (3)
C4—C5—C6—C1	0.2 (5)	C4—C5—C10—C11	−34.9 (3)
C10—C5—C6—C1	178.2 (3)	O1—C9—C10—C5	168.1 (2)
C2—C1—C6—C5	−0.9 (5)	C8—C9—C10—C5	−69.6 (3)
C3—C4—C7—C8	106.6 (4)	O1—C9—C10—C11	−87.6 (3)
C5—C4—C7—C8	−71.0 (3)	C8—C9—C10—C11	34.7 (3)
C3—C4—C7—C11	−147.6 (3)	C4—C7—C11—C10	−53.4 (2)
C5—C4—C7—C11	34.8 (3)	C8—C7—C11—C10	57.6 (2)
C4—C7—C8—O2	−176.5 (2)	C5—C10—C11—C7	53.4 (2)
C11—C7—C8—O2	78.7 (3)	C9—C10—C11—C7	−56.4 (2)
C4—C7—C8—C9	67.4 (3)		

### Hydrogen-bond geometry (Å, °)

**Table:**

*D*—H···*A*	*D*—H	H···*A*	*D*···*A*	*D*—H···*A*
O1—H1A···O2	0.85	2.16	2.578 (3)	110
O1—H1A···O2^i^	0.85	2.34	2.818 (3)	116
O2—H2A···O1^ii^	0.82	1.90	2.714 (3)	176

Symmetry codes: (i) −*x*+1, −*y*, −*z*+1; (ii) *x*−1/2, −*y*+1/2, −*z*+1.
